# Analysis of autophagy gene polymorphisms in Spanish patients with head and neck squamous cell carcinoma

**DOI:** 10.1038/s41598-017-07270-0

**Published:** 2017-07-31

**Authors:** Javier Fernández-Mateos, Raquel Seijas-Tamayo, Juan Carlos Adansa Klain, Miguel Pastor Borgoñón, Elisabeth Pérez-Ruiz, Ricard Mesía, Elvira del Barco, Carmen Salvador Coloma, Antonio Rueda Dominguez, Javier Caballero Daroqui, Encarnación Fernández Ruiz, Juan Jesús Cruz-Hernández, Rogelio González-Sarmiento

**Affiliations:** 1 Medical Oncology Service, University Hospital of Salamanca-IBSAL, Salamanca, 37007 Spain; 2 Biomedical Research Institute of Salamanca (IBSAL), SACYL-University of Salamanca-CSIC, Salamanca, 37007 Spain; 3 Molecular Medicine Unit- IBSAL, Department of Medicine, University of Salamanca, 37007 Salamanca, Spain; 4 Institute of Molecular and Cellular Biology of Cancer (IBMCC), University of Salamanca-CSIC, Salamanca, 37007 Spain; 5 Medical Oncology Service, Hospital Universitario Politécnico La Fe, Valencia, 46026 Spain; 6 Division of Medical Oncology, Oncology department, Agencia Sanitaria Hospital Costa del Sol de Marbella, 29603 Marbella, Spain; 7 Medical Oncology Department, Universitat de Barcelona, IDIBELL, Institut Català d’Oncologia, L’Hospitalet de Llobregat, Barcelona, 08908 Spain; 8 Otolaryngology Department, Agencia Sanitaria Hospital Costa del Sol de Marbella, Marbella, 29603 Spain

## Abstract

Head and neck squamous cell carcinoma (HNSCC) is the sixth cancer on incidence worldwide. Tobacco and alcohol consumption are the most classical risk factors associated with its development. Autophagy process has a dual effect both in tumourigenesis and tumour suppressing activity. To investigate the importance of this pathway in HNSCC susceptibility, a risk factor matched case-control association study was performed with four candidate polymorphisms in autophagy genes (ATG2B, ATG5, ATG10, ATG16L1). We found an association between the variant in ATG10 rs1864183 and a higher susceptibility to develop laryngeal cancer, ATG2B rs3759601 and pharyngeal cancer and ATG16L1 rs2241880 and oral carcinoma. ATG5 rs2245214 SNP was not associated with any location. Overall, our results indicate the importance of the autophagy pathway in the susceptibility of head and neck squamous cell carcinoma and demonstrate the heterogeneity between its locations encompassed under a single terminology.

## Introduction

Head and neck cancer (HNC) groups a set of different tumours located in the upper aero-digestive via. It includes tumours located in the lips, oral cavity, pharynx (nasopharynx, oropharynx and hypopharynx) as well as larynx, salivary glands and thyroid glands among others^1^. It is the sixth cancer type on incidence worldwide. Approximately 600.000 new cases are diagnosed each year and only 40–50% reach the five-years survival rate^2^ causing an annual death of 271.000 patients^3, 4^. Not all HNC present similar histology, prevailing in 90% of cases the squamous cell carcinomas which initiate in the mucosa^1^.

Classic factors associated to the development of head and neck squamous cell carcinoma (HNSCC) are tobacco and alcohol consumption. At least 75% HNSCC are attributable to the combination of both carcinogens^5, 6^. Moreover, different epidemiological studies have revealed the existence of other related factors, both environmental and genetic. In the last years the viral aetiology has been implicated in the development of HNSCC. This is the case of Epstein-Barr virus (EBV) in nasopharynx and the human papillomavirus (HPV), mainly subtype 16, in oral cavity and oropharynx tumours^7^. However, the carcinogenesis procedure triggered by viral infection identifies a totally different entity than the one produced by tobacco and alcohol^8, 9^. On the other hand, the observation of familial aggregations in HNSCC suggests the existence of genetic predisposition factors. Lots of case-control studies have determined this genetic susceptibility, increasing the risk between 2–4 times for first grade HNSCC patients family^10^.

Aero digestive tract carcinogenesis involves altered carcinogen metabolism, a modified DNA repair, cell cycle disruption and deregulation of pathways implicated in immunity, inflammation and cellular components degradation^11^. Allelic variants of genes implicated in essential cellular pathways play a very important role in tumour development as well as in treatment response. Polymorphism is defined as that mutation or variant which is found in at least 1% of the general population. Single Nucleotide Polymorphism (SNP) is the most abundant form of genetic variation^11^.

Macro-autophagy is the catabolic process of damaged organelles or protein recycling under nutrient starvation or stress. It starts with the double-membrane autophagosome formation and finishes with a fusion with the lysosomes to form the autophagolysosome which contains hydrolases for the degradation of the contents. This autophagosome complex is synthesised by autophagy-related genes (ATG)^12^. Autophagy takes part into both the initiation and prevention of cancer, and its function can be altered during tumour progression^13^. Although autophagy has a suppressing tumour activity, it is also involved in tumourigenesis by inhibiting cellular death and increasing drug resistance. It participates in important pathways connected to carcinogenesis as well as immune response, inflammation and genome stability^14^. However the precise mechanisms that involve autophagy in cancer are not yet defined^15^. In HNSCC, autophagy mechanisms are still unknown and they can symbolize an important area for future research^16^.

To achieve our aim a candidate gene analysis was performed to study SNPs in autophagy genes: ATG2B, ATG5, ATG10, ATG16L1 (Table 1) that could be associated to the risk to suffer HNSCC in a Spanish population. This association study was performed with a control group, selecting a cohort of subjects matched in gender, age and the two most important environmental factors involved in the development of HNSCC, tobacco and alcohol consumption, avoiding confounding variables and considering genetic background on its own.

**Table 1:** Autophagy polymorphisms analysed in the study.

Gene	SNP ID	Base change	Protein change	Chr. location	Assay ID	HWE*
**ATG2B**	rs3759601	C > G	Q1383E	14:96311131	c_9690166_10	>0.05
**ATG5**	rs2245214	C > G	Intronic	6:106214866	c_3001905_20	>0.05
**ATG10**	rs1864183	C > T	T212M	5:82253397	c_11953871_20	>0.05
**ATG16L1**	rs2241880	T > C	T300A	2:233274722	c_9095577_20	>0.05

^*^Hardy-Weinberg equilibrium (HWE) calculated in the control group.

## Results

A total of 450 patients distributed in 213 cases of larynx carcinoma, 165 of pharynx carcinoma and 72 of oral cavity were included in the study. The descriptive study of the global analysis by location showed some statistical differences between sex, age, tobacco and alcohol intake (Table 2). For this reason, SNPs analysis was calculated with an adjustment for these variables in the different locations.

**Table 2:** Descriptive case-control study.

Characteristics	PATIENTS N = 450		CONTROLS N = 253		P-value	LARYNX N = 213		P-value	PHARYNX N = 165		P-value	ORAL CAVITY N = 72		P-value
	N	%	N	%		N	%		N	%		N	%	
Age (years)	61.97 ± 9.242		52.18 ± 12.752		**0.000**	62.96 ± 8.987		**0.000**	61.00 ± 9.086		**0.000**	61.29 ± 10.232		**0.000**
**Sex**														
Female	52	11.6	130	51.4	**0.000**	13	6.1	**0.000**	23	13.9	**0.000**	16	22.2	**0.000**
Male	398	88.4	123	48.6		200	93.9		142	86.1		56	77.8	
**Tobacco smoking**														
Never	22	4.9	23	9.1	**0.030**	7	3.3	**0.000**	8	4.8	**0.013**	7	9.7	0.162
< 20 PPY	62	13.8	72	28.5		20	9.4		30	18.2		12	16.7	
> 20 PPY	352	78.2	146	57.7		180	84.5		121	73.3		51	70.8	
Missing	14	3.1	12	4.7		6	2.8		6	3.6		2	2.8	
Packs per year	57.00 ± 36.512		31.88 ± 28.861		**0.000**	61.17 ± 35.498		**0.000**	54.91 ± 36.947		**0.000**	49.55 ± 37.366		**0.000**
**Alcohol drinking**														
Never	105	23.3	153	60.5	**0.000**	53	24.9	**0.000**	27	16.4	**0.000**	25	34.7	**0.000**
< 14 SDU/week	94	20.9	46	18.2		44	20.7		31	18.8		19	26.4	
> 14 SDU/week	238	52.9	48	19.0		114	53.5		96	58.2		28	38.9	
Missing	13	2.9	6	2.4		2	0.9		11	6.7		0	0	
SDU/week	30.07 ± 39.349		9.02 ± 21.213		**0.000**	27.81 ± 37.008		**0.000**	36.27 ± 40.710		**0.000**	23.43 ± 41.553		**0.000**

P-values related to controls. Statistically significant results in bold.

The global study of susceptibility in laryngeal cancer (Table 3) showed an association between the heterozygote genotype of ATG2B rs3759601 and a lower risk to develop laryngeal squamous cell carcinoma, p = 0.049 OR = 0.607 (0.369–0.999). Moreover, although not statistically significant a tendency in ATG10 rs1864183 was found. The heterozygous genotype had a close relationship with an increased risk to develop laryngeal cancer (p = 0.059, OR = 1.648) (Table 3).

**Table 3:** Comparative results in selected ATG polymorphism distribution in laryngeal, pharyngeal and oral cavity cancer related to controls.

	Genotype	Control		Larynx				Pharynx				Oral cavity			
		N	%	N	%	P-value	OR (CI 95%)	N	%	P-value	OR (CI 95%)	N	%	P-value	OR (CI 95%)
**ATG2B rs3759601**	CC	106	41.9	98	46.0	/	1.00	63	38.2	/	1.00	28	38.9	/	1.00
	CG	119	47.0	88	41.3	**0.049**	**0.607 (0.369–0.999)**	80	48.5	0.749	1.091 (0.640–1.858)	36	50.0	0.720	1.120 (0.603–2.080)
	GG	28	11.1	27	12.7	0.921	1.041 (0.472–2.296)	22	13.3	**0.016**	**2.613 (1.200–5.690)**	8	11.1	0.522	1.391 (0.506–3.821)
**Recessive**	CC + CG	225	88.9	186	87.3	/	1.00	143	86.7	/	1.00	64	88.9	/	1.00
	GG	28	11.1	27	12.7	0.444	1.339 (0.635–2.825)	22	13.3	**0.013**	**2.493 (1.212–5.129)**	8	11.1	0.583	1.301 (0.508–3.333)
**Dominant**	CC	106	41.9	98	46.0	/	1.00	63	38.2	/	1.00	28	38.9	/	1.00
	CG + GG	147	58.1	115	54.0	0.103	0.675 (0.421–1.083)	102	61.8	0.282	1.317 (0.789–2.175)	44	61.1	0.630	1.158 (0.637–2.106)
**ATG5 rs2245214**	CC	104	41.1	82	38.5	/	1.00	72	43.6	/	1.00	31	43.1	/	1.00
	CG	124	49.0	105	49.3	0.369	1.256 (0.763–2.068)	79	47.9	0.624	0.879 (0.524–1.474)	31	43.1	0.348	0.744 (0.401–1.380)
	GG	25	9.9	26	12.2	0.274	1.551 (0.707–3.401)	14	8.5	0.638	0.810 (0.337–1.946)	10	13.9	0.611	1.272 (0.503–3.216)
**Recessive**	CC + CG	228	90.1	187	87.8	/	1.00	151	91.5	/	1.00	62	86.1	/	1.00
	GG	25	9.9	26	12.2	0.406	1.364 (0.656–2.837)	14	8.5	0.743	0.870 (0.380–1.993)	10	13.9	0.380	1.480 (0.617–3.552)
**Dominant**	CC	104	41.1	82	38.5	/	1.00	72	43.6	/	1.00	31	43.1	/	1.00
	CG + GG	149	58.9	131	61.5	0.272	1.307 (0.811–2.107)	93	56.4	0.574	0.867 (0.527–1.426)	41	56.9	0.531	0.831 (0.465–1.485)
**ATG10 rs1864183**	CC	93	36.8	70	32.9	/	1.00	46	27.9	/	1.00	27	37.5	/	1.00
	CT	115	45.4	116	54.4	0.059	1.648 (0.981–2.770)	86	52.1	0.127	1.537 (0.885–2.670)	34	47.2	0.452	1.274 (0.678–2.392)
	TT	45	17.8	27	12.7	0.946	1.026 (0.493–2.133)	33	20.0	0.201	1.594 (0.780–3.260)	11	15.3	0.875	0.931 (0.384–2.257)
**Recessive**	CC + CT	208	82.2	186	87.3	/	1.00	132	80.0	/	1.00	61	84.7	/	1.00
	TT	45	17.8	27	12.7	0.415	0.760 (0.392–1.472)	33	20.0	0.517	1.232 (0.656–2.312)	11	15.3	0.612	0.812 (0.363–1.817)
**Dominant**	CC	93	36.8	70	32.9	/	1.00	46	27.9	/	1.00	27	37.5	/	1.00
	CT + TT	160	63.2	143	67.1	0.118	1.484 (0.905–2.434)	119	72.1	0.100	1.552 (0.920–2.618)	45	62.5	0.587	1.180 (0.649–2.147)
**ATG16L1 rs2241880**	TT	72	28.5	58	27.2	/	1.00	44	26.7	/	1.00	18	25.0	/	1.00
	TC	130	51.3	108	50.7	0.597	1.157 (0.674–1.988)	81	49.1	0.551	1.194 (0.667–2.137)	31	43.1	0.860	1.066 (0.524–2.168)
	CC	51	20.2	47	22.1	0.312	1.414 (0.722–2.769)	40	24.2	0.166	1.647 (0.813–3.335)	23	31.9	**0.039**	**2.304 (1.043–5.093)**
**Recessive**	TT + TC	202	79.8	166	77.9	/	1.00	125	75.8	/	1.00	49	68.1	/	1.00
	CC	51	20.2	47	22.1	0.389	1.288 (0.724–2.292)	40	24.2	0.205	1.469 (0.810–2.666)	23	31.9	**0.017**	**2.214 (1.150–4.263)**
**Dominant**	TT	72	28.5	58	27.2	/	1.00	44	26.7	/	1.00	18	25.0	/	1.00
	TC + CC	181	71.5	155	72.8	0.436	1.226 (0.735–2.046)	121	73.3	0.332	1.313 (0.758–2.276)	54	75.0	0.321	1.393 (0.724–2.682)

P value & OR adjusted by sex, age, packs per year and SDU per week. Statistically significant results in bold.

Analysis in pharyngeal squamous cell carcinoma showed that carriers of GG genotype in the SNP ATG2B rs3759601 had an increased risk to develop this tumour, both in the codominant and the recessive model, p = 0.013 OR = 2.493 (1.212–5.129) (Table 3). No other associations were found in the rest of SNPs between cases and controls.

ATG16L1 rs2241880 was unequally distributed in oral cavity cancer (Table 3). Patients with the less common allele C had higher risk to suffer from oral cavity cancer in our sample, p = 0.017 in recessive model, OR = 2.214 (1.150–4.263).

Due to the great significant differences in all the variables between groups, a second analysis was proposed by the Propensity Score method (PS). After its application we have totally paired 126 larynx, 100 pharynx and 70 oral cavity tumours according to sex, packs of tobacco per year (PPY) and standard drink units per week (SDU/week) with their specific control groups (Table 4). This method allowed us to corroborate the previous analysis avoiding the possible confounding variables. Quantitative age was also included as an adjustment variable in the logistic regression analysis of the laryngeal susceptibility study due to the significant differences between groups in the ANOVA test (p-value < 0.05) (Table 4). Because of pharyngeal and oral cavity carcinomas were paired by age, adjustment by quantitative age was not necessary (Table 4).

**Table 4:** Descriptive case-control study matched by the Propensity Score method.

Characteristics	LARYNX N = 126		CONTROL N = 126		P-value	PHARYNX N = 100		CONTROL N = 100		P-value	ORAL CAVITY N = 70		CONTROL N = 70		P-value
	N	%	N	%		N	%	N	%		N	%	N	%	
Age (years)	63.02 ± 8.566		56.30 ± 12.803		**0.000**	59.96 ± 8.41		59.52 ± 10.044		0.742	60.92 ± 10.008		62.24 ± 8.88		0.412
**Sex**															
Female	13	10.3	13	10.3	1.000	20	20.0	22	22.0	0.728	16	22.9	17	22.9	1.000
Male	113	89.7	113	89.7		80	80.0	78	78.0		54	77.1	54	77.1	
**Tobacco smoking**															
Never	7	5.5	7	5.5	0.944	7	7.0	8	8.0	0.943	7	10.0	7	10.0	1.000
<20 PPY	20	15.9	22	17.5		22	22.0	23	23.0		12	17.1	12	17.1	
>20 PPY	99	78.6	97	77.0		71	71.0	69	69.0		51	72.9	51	72.9	
Missing	0	0	0	0		0	0	0	0		0	0	0	0	
**Alcohol drinking**															
Never	53	42.1	51	40.5	0.904	26	26.0	27	27.0	0.985	23	32.9	23	32.9	1.000
<14 SDU/week	28	22.2	31	24.6		30	30.0	30	30.0		19	27.1	19	27.1	
>14 SDU/week	45	35.7	44	34.9		44	44.0	43	43.0		28	40.0	28	40.0	
Missing	0	0	0	0		0	0	0	0		0	0	0	0	

P-values related to controls. Statistically significant results in bold.

Once again, ATG2B rs3759601 heterozygote genotype was associated with a lower risk to develop laryngeal cancer p = 0.028 OR = 0.535 (0.307–0.935) (Table 5). Although not statistically significant in the previous analysis (p = 0.059), we found a similar result in ATG10 rs1864183 and a higher risk to develop laryngeal cancer in patients carrying the T allele, p = 0.026 OR = 1.888 (1.078–3.308) in the dominant model.

**Table 5:** Comparative results in selected ATG polymorphism distribution in risk factor-matched laryngeal cancer and controls.

	Genotype	Larynx		Control		P-value*	OR (CI 95%)
		N	%	N	%		
**ATG2B rs3759601**	CC	59	46.8	46	36.5	/	1.00
	CG	52	41.3	69	54.8	**0.028**	**0.535 (0.307–0.935)**
	GG	15	11.9	11	8.7	0.904	1.058 (0.423–2.644)
**Recessive**	CC + CG	111	88.1	115	91.3	/	1.00
	GG	15	11.9	11	8.7	0.375	1.479 (0.624–3.506)
**Dominant**	CC	59	46.8	46	36.5	/	1.00
	CG + GG	67	53.2	80	63.5	0.063	0.604 (0.355–1.028)
**ATG5 rs2245214**	CC	47	37.3	49	38.9	/	1.00
	CG	63	50.0	66	52.4	0.725	1.105 (0.633–1.931)
	GG	16	12.7	11	8.7	0.269	1.662 (0.675–4.089)
**Recessive**	CC + CG	110	87.3	115	91.3	/	1.00
	GG	16	12.7	11	8.7	0.294	1.567 (0.677–3.627)
**Dominant**	CC	47	37.3	49	38.9	/	1.00
	CG + GG	79	62.7	77	61.1	0.533	1.186 (0.693–2.031)
**ATG10 rs1864183**	CC	38	30.2	50	39.7	/	1.00
	CT	70	55.5	58	46.0	**0.020**	**2.004 (1.114–3.608)**
	TT	18	14.3	18	14.3	0.312	1.531 (0.671–3.494)
**Recessive**	CC + CT	108	85.7	108	85.7	/	1.00
	TT	18	14.3	18	14.3	0.985	1.007 (0.481–2.110)
**Dominant**	CC	38	30.2	50	39.7	/	1.00
	CT + TT	88	69.8	76	60.3	**0.026**	**1.888 (1.078–3.308)**
**ATG16L1 rs2241880**	TT	40	31.7	40	31.7	/	1.00
	TC	62	49.3	66	52.4	0.930	1.027 (0.570–1.848)
	CC	24	19.0	20	15.9	0.415	1.382 (0.635–3.010)
**Recessive**	TT + TC	102	81.0	106	84.1	/	1.00
	CC	24	19.0	20	15.9	0.381	1.359 (0.684–2.701)
**Dominant**	TT	40	31.7	40	31.7	/	1.00
	TC + CC	86	68.3	86	68.3	0.723	1.106 (0.633–1.935)

^*^P value & OR adjusted by age. Statistically significant results in bold.

PS method corroborated the result in the previous analyses finding an association between ATG2B rs3759601 G allele and a higher risk to suffer from pharynx cancer (p = 0.035, OR = 2.721 (1.075–6.887)) (Table 6).

**Table 6:** Comparative results in selected ATG polymorphism distribution in risk factor-matched pharyngeal cancer and controls.

	Genotype	Pharynx		Control		P-value	OR (CI 95%)
		N	%	N	%		
**ATG2B rs3759601**	CC	35	35.0	44	44.0	/	1.00
	CG	48	48.0	49	49.0	0.494	1.231 (0.678–2.235)
	GG	17	17.0	7	7.0	**0.026**	**3.053 (1.139–8.182)**
**Recessive**	CC + CG	83	83.0	93	93.0	/	1.00
	GG	17	17.0	7	7.0	**0.035**	**2.721 (1.075–6.887)**
**Dominant**	CC	35	35.0	44	44.0	/	1.00
	CG + GG	65	65.0	56	56.0	0.194	1.459 (0.825–2.580)
**ATG5 rs2245214**	CC	47	47.0	39	39.0	/	1.00
	CG	45	45.0	52	52.0	0.265	0.718 (0.401–1.286)
	GG	8	8.0	9	9.0	0.567	0.738 (0.260–2.092)
**Recessive**	CC + CG	92	92.0	91	91.0	/	1.00
	GG	8	8.0	9	9.0	0.800	0.879 (0.325–2.379)
**Dominant**	CC	47	47.0	39	39.0	/	1.00
	CG + GG	53	53.0	61	61.0	0.254	0.721 (0.411–1.265)
**ATG10 rs1864183**	CC	30	30.0	38	38.0	/	1.00
	CT	51	51.0	47	47.0	0.316	1.374 (0.738–2.559)
	TT	19	19.0	15	15.0	0.264	1.604 (0.700–3.676)
**Recessive**	CC + CT	81	81.0	85	85.0	/	1.00
	TT	19	19.0	15	15.0	0.452	1.329 (0.633–2.792)
**Dominant**	CC	30	30.0	38	38.0	/	1.00
	CT + TT	70	70.0	62	62.0	0.233	1.430 (0.794–2.575)
**ATG16L1 rs2241880**	TT	27	27.0	34	34.0	/	1.00
	TC	48	48.0	49	49.0	0.522	1.234 (0.648–2.347)
	CC	25	25.0	17	17.0	0.130	1.852 (0.835–4.108)
**Recessive**	TT + TC	75	75.0	83	83.0	/	1.00
	CC	25	25.0	17	17.0	0.167	1.627 (0.816–3.247)
**Dominant**	TT	27	27.0	34	34.0	/	1.00
	TC + CC	73	73.0	66	66.0	0.283	1.393 (0.761–2.551)

Statistically significant results in bold.

Finally, ATG16L1 rs2241880 CC genotypes still being associated with a higher risk to develop oral carcinoma after the PS application, p = 0.047 OR = 2.299(1.010–5.230) (Table 7).

**Table 7:** Comparative results in selected ATG polymorphism distribution in risk factor-matched oral cavity cancer and controls.

	Genotype	Oral cavity		Control		P-value	OR (CI 95%)
		N	%	N	%		
**ATG2B rs3759601**	CC	27	38.6	27	38.6	/	1.00
	CG	36	51.4	39	55.7	0.823	0.923 (0.458–1.859)
	GG	7	10.0	4	5.7	0.413	1.750 (0.459–6.679)
**Recessive**	CC + CG	63	90.0	66	94.3	/	1.00
	GG	7	10.0	4	5.7	0.352	1.833 (0.512–6.568)
**Dominant**	CC	27	38.6	27	38.6	/	1.00
	CG + GG	43	61.4	43	61.4	1.000	1.000 (0.506–1.975)
**ATG5 rs2245214**	CC	31	44.3	25	35.7	/	1.00
	CG	31	44.3	35	50.0	0.356	0.714 (0.349–1.460)
	GG	8	11.4	10	14.3	0.421	0.645 (0.222–1.878)
**Recessive**	CC + CG	62	88.6	60	85.7	/	1.00
	GG	8	11.4	10	14.3	0.614	0.774 (0.286–2.094)
**Dominant**	CC	31	44.3	25	35.7	/	1.00
	CG + GG	39	55.7	45	64.3	0.301	0.699 (0.354–1.379)
**ATG10 rs1864183**	CC	26	37.1	26	37.1	/	1.00
	CT	33	47.2	33	47.2	1.000	1.000 (0.483–2.069)
	TT	11	15.7	11	15.7	1.000	1.000 (0.369–2.710)
**Recessive**	CC + CT	59	84.3	59	84.3	/	1.00
	TT	11	15.7	11	15.7	1.000	1.000 (0.402–2.485)
**Dominant**	CC	26	37.1	26	37.1	/	1.00
	CT + TT	44	62.9	44	62.9	1.000	1.000 (0.504–1.985)
**ATG16L1 rs2241880**	TT	18	25.7	26	37.1	/	1.00
	TC	31	44.3	33	47.2	0.441	1.357 (0.625–2.947)
	CC	21	30.0	11	15.7	**0.035**	**2.758 (1.072–7.096)**
**Recessive**	TT + TC	49	70.0	59	84.3	/	1.00
	CC	21	30.0	11	15.7	**0.047**	**2.299 (1.010–5.230)**
**Dominant**	TT	18	25.7	26	37.1	/	1.00
	TC + CC	52	74.3	44	62.9	0.147	1.707 (0.829–3.517)

Statistically significant results in bold.

## Discussion

HNSCC is consequence of genetic and environmental factors, mainly tobacco smoking and alcohol consumption. Autophagy is a complex pathway, modulated by different molecular mechanisms with an important interest in HNSCC development^16^. To show the possible association of polymorphisms in autophagy genes and the susceptibility to suffer these tumours, a multicentre case-control study of head and neck squamous cell carcinoma was performed. Four polymorphisms were selected in ATG genes involved in phagosome generation. This was the case of the exonic missense polymorphisms ATG2B rs3759601, ATG16L1 rs2241880 and ATG10 rs1864183, and the intronic mutation in ATG5 rs2245214 which involves changes in the recognition sites for SRp40 transcription factor. ATG5, ATG10 and ATG16L1 code for proteins that form the Atg5-Atg12-Atg16L1 conjugation complex^17^, while Atg2B is necessary for closure of isolation membranes of autophagosomes^18^.

Analysis of laryngeal cancer showed an association between the less common allele genotypes (CT + TT) in ATG10 rs1864183 and a higher risk to develop it. It has been described that a lower expression of autophagy genes (ATG) accelerate tumour development due to a diminution in autophagy process^19^. ATG10 rs1864183 C > T variant in exon 4 leads a catalytic change in the protein (Thr212Met) which causes a dysregulation in the autophagosome formation and a higher risk to develop breast cancer^20^. In this situation the cell cannot degrade a damaged organ, collecting damaging substances that cause an increase in DNA damage and carcinogenesis. Although this polymorphism has never been studied in HNSCC, this result indicates the importance of the autophagy pathway in laryngeal tumour. We could hypothesize that less common allele genotypes (CT + TT) could be related with a lower autophagy and accumulation of DNA damage, related with a higher risk to develop laryngeal squamous cell carcinoma.

Though only associated in ATG2B rs3759601 heterozygosity (CG), there was a statistically significant result related with a lower risk of develop laryngeal cancer. However this result is difficult to explain due to its non-significance in dominant models. Nevertheless, there was a positive association between the homozygous GG genotype in the same polymorphism and an increase risk to suffer from pharyngeal squamous cell carcinoma. In mammals, there are two ATG2 genes which are functionally redundant^21^. Atg2B is an essential protein in the autophagy process due to it is essential for the autophagosome and lipid droplets formation^19, 22^. Mutations in ATG2B gene have been associated with colorectal and gastric cancer^14^. Atg2B rs3759601 C > G SNP in exon 25 produces a protein change p.Gln1382Glu which could result in diminished autophagy and a higher risk to suffer pharyngeal cancer in our sample.

We did not find any significant result in the intronic ATG5 rs2245214 SNP distribution and HNSCC susceptibility. This result can be related with the position of this polymorphism in the intronic region 6 of the ATG5 gene and the consequence of ineffective change in the protein function.

Finally, we found an association in the distribution of CC genotypes in the dominant and recessive models of ATG16L1 rs2241880 polymorphism and a higher risk to suffer from oral cavity squamous cell carcinoma. Autophagy-related 16-like 1 (ATG16L1) gene is a central adaptor in Atg5-Atg12-Atg16L1 complex formation and elongation of the autophagosome^23^. ATG16L1 variant rs2241880, a nonsynonymous 898 T > C polymorphism that encodes a threonine-to-alanine change (T300A), is associated with a decreased autophagy in Crohn's disease and higher inflammation^23^. In these studies CC genotype increases the secretion of TNF-α and IL-1β promoting a higher inflammation^23^. It has been also described that T300A variant enhances ATG16L1 cleavage by caspase 3, resulting in defective autophagy^24^ and chronic inflammatory state which increase Crohn´s disease susceptibility^25^ and colorectal cancer^26^. Likewise, it is known that ATG16L1-T300A SNP shows reduced affinity to bind the ATG16L1-binding motif and higher caspase-3 processing, causing a defective autophagy process^27^. Our results showed that CC genotypes were associated with an increased susceptibility to develop oral cavity squamous cell carcinoma maybe due to lower autophagy and a higher inflammation, a very important pathway implied in the aetiology of this tumour^28^.

In conclusion, this study provides evidence of the putative role of some polymorphisms in autophagy genes as a genetic susceptibility factor in head and neck squamous cell carcinogenesis. This is the first autophagy susceptibility study in which cases and controls are matched by their risk factors, only taking into account their genetic background. Our finding emphasize the importance of autophagy in these tumours, the same as the heterogeneity between locations include under the same term of head and neck cancer. Additional studies in larger groups should be done and would be necessary to confirm our results.

## Material and Methods

### Study design

The data presented here is part of a multicentre study of three years of duration coordinated by the Medical Oncology Department of the University Hospital of Salamanca with the collaboration of 20 Spanish hospitals, all of them belonging to the Spanish Head & Neck Cancer Cooperative Group (TTCC).

The recruitment period extended from January 2012 to December 2014. The inclusion criteria were: adults diagnosed of HPV negative squamous cell carcinoma of larynx, pharynx or oral cavity. They were recruited in different Spanish hospitals that participate in the project after signing a written informed consent designed for this project according to local rules. The protocol of TTCC-2010–05 was initially approved by the TTCC Executive Committee, and then by the local institutional review board of University Hospital of Salamanca, according to country regulations. The research was conducted in full accordance with the ethical standards laid down in the 1964 Declaration of Helsinki and its later amendments and was consistent with Good Clinical Practice guidelines and the applicable local regulatory requirements.

455 patients diagnosed of HNSCC were included in the study. Controls were hospitalized patients without personal or familial history of cancer trying to be paired with cases by age, sex, smoking and alcoholism habit. They were recruited in different departments of the 20 hospitals. The initial sample size calculated for the control group was the same than the number of patients included in the study. However, this size was not reached due to the restricted inclusion criteria, so finally only 259 controls were included.

The information into socio-demographic and data informed by patient questionnaires were collected by auto-application, being supervised by the member of the research team with the objective of correct filled. Tumour clinic-pathological data were collected by oncologists following the TNM grading system reported by the American Joint Committee on Cancer (AJCC). All data were treated with the security measures establish in compliance with the Protection of Personal Data Organic Law 15/1999, 13^th^ December, and safe-keeping by the University Hospital of Salamanca in its specific hospital server. Global study recruitment procedures and data collection have been previously described^29^.

### DNA isolation and genotyping

DNA was extracted from leukocytes of peripheral blood tube by phenol-chloroform method. Four polymorphisms in important ATG genes (ATG2B rs3759601, ATG5 rs2245214, ATG10 rs1864183 and ATG16L1 rs2241880) were selected according to the following criteria: previously described association with illness susceptibility, >5% minor allele frequency in Caucasian population and published evidence of functionality. Genotyping of selected polymorphisms (Table 1) were analyzed by the allelic discrimination assay by TaqMan® probes (Applied biosystems), with specific oligonucleotides to amplify the polymorphic sequences and two labelled probes with the fluorochrome VIC and FAM to detect both alleles of each polymorphism. The reaction was performed using the specific PCR Master Mix in the Step-One Plus Real-Time PCR system (Applied biosystems)^30^. To ensure the reproducibility, a 5% of random samples were re-genotyping. A total of 11 samples (5 patients and 6 controls) cannot be amplified due to low DNA quality rate and were excluded of the study.

### Statistical analysis

Control group was tested for assumption of the Hardy-Weinberg equilibrium (HWE) by chi-squared test for each polymorphism (Table 1). The association between the different clinical and molecular variables was analyzed by cross tabs and the *X* ^2^ test of Pearson. The Odds ratio (OR) and 95% confidence intervals were calculated by a logistic regression analysis. It was considered the existence of statistically significant differences where the P-value was < 0.05. These analyses were performed with the statistical software SPSS v.21.0 (IBM).

Because of the lower inclusion of matched controls, the statistical analysis was realized in two different ways. Firstly, patients were stratified according to its location (larynx, pharynx and oral cavity) comparing with the global control group (Table 2). To take into account the possible confounding variables, it was made a statistical adjustment for sex, and the continuous variables of age, packs of tobacco per year (PPY) and standard drink units of alcohol per week (SDU/week).

Secondly, we used the Propensity Score method (PS), a statistical term applied to the potent matching technique to equate groups in a cohort study^31^. Through a logistic regression analysis introducing the confounders as predictive variables, the method provides a numeric probability of each predictor group^32^. PS allows to pair the cases with the controls through the selection of a control sample with the same characteristics than patients regarding sex, tobacco and alcohol consumption. In this way both groups were are matched according to: packs of tobacco consumed per year (PPY): no smokers, <20PPY and >20PPY, standard drink units of alcohol per week (SDU/week): <14 SDU/week and >14 SDU/week and sex (Table 4). As the Propensity Score method did not include the age of the individuals, in the second analysis age was introduced in the logistic regression as adjustment variable only in laryngeal carcinoma where this variable was statistically significant (p > 0.05 by ANOVA test).
